# An Update on Assessment, Therapeutic Management, and Patents on Insomnia

**DOI:** 10.1155/2021/6068952

**Published:** 2021-10-18

**Authors:** Amit Porwal, Yogesh Chand Yadav, Kamla Pathak, Ramakant Yadav

**Affiliations:** ^1^Faculty of Pharmacy, Uttar Pradesh University of Medical Sciences, Saifai, Etawah, 206130 Uttar Pradesh, India; ^2^Faculty of Medical Sciences, Uttar Pradesh University of Medical Sciences, Saifai, Etawah, 206130 Uttar Pradesh, India

## Abstract

Insomnia is an ordinary situation related to noticeable disability in function and quality of life, mental and actual sickness, and mishappenings. It represents more than 5.5 million appointments to family doctors every year. Nonetheless, the ratio of insomniacs who are treated keeps on being low, demonstrating the requirement for proceeding with advancement and dispersal of effective treatments. Accordingly, it becomes significant to provide a compelling treatment for clinical practice. It indicates a need for the determination of various critical viewpoints for the evaluation of insomnia along with various accessible alternatives for treatment. These alternatives incorporate both nonpharmacological therapy, specifically cognitive behavioural therapy for insomnia, and a number of pharmacological treatments like orexin antagonists, “z-drugs,” benzodiazepines, selective histamine H1 antagonists, nonselective antihistamines, melatonin receptor agonists, antipsychotics, antidepressants, and anticonvulsants. Besides in individuals whose insomnia is due to restless leg syndrome, depression/mood disorder, or/and circadian disturbance, there is insignificant proof favouring the effectiveness of different prescriptions for the treatment of insomnia though they are widely used. Other pharmacological agents producing sedation should be prescribed with care for insomnia therapy because of greater risk of next-day sleepiness along with known adverse effects and toxicities. This review is also aimed at providing an update on various patents on dosage forms containing drugs for insomnia therapy.

## 1. Introduction

Insomnia is one of the most widely recognized issues experienced by the medical practitioners, accounting for more than 5.5 million prescriptions yearly [1]. Insomnia has been defined by the American Academy of Sleep Medicine to be the individual observation of trouble related to onset of sleep, time span, consolidation, or quality that come about regardless of sufficient time for sleep and that outcomes in some type of mental and physical unfitness during daytime [2–5]. Insomnia is a typical circumstance with prevalence point of approximately 10% in general population [6–9]. It is related to at least one of the following symptoms: trouble in sleep initiation (initial insomnia or sleep-onset insomnia), trouble in maintaining sleep (middle insomnia or sleep-maintenance insomnia), and early-morning arousing along with problem to get back to sleep (late insomnia) [4, 10].

There is a tremendous rise in the number of patients visiting physicians for insomnia from 5.3 million in 1999 to 208 million in 2010 [1]. The possibility of females experiencing insomnia is greater as compared to males, and they are two times as liable to be diagnosed with sleep disorders [8, 11]. In case of females, the hormonal changes, generally in the third trimester of pregnancy and postmenopausal changes, are also responsible for increasing the frequency of insomnia [8]. Though insomnia is especially common in elderly individuals, with indications found in almost 65% of people 65 years or older, it can occur at any age [12, 13]. Patients with concomitant diseases like respiratory problems, cardiovascular diseases, painful conditions, and neurological illness are at greater risk [14]. Population studies propose that it is highly predominant in individuals who are separated, unemployed, have encountered the demise of a family member, widowed, divorced, or of lower financial status [15–17]. It is also highly reported in individuals withdrawing from narcotics or alcohol.

In most of the instances, insomnia occurs concomitantly with physical or psychiatric problems. The relation between insomnia and the above conditions are complicated and at times bidirectional [18–21]. Indeed, insomnia can be a risk factor for nervousness, major depression, diabetes, hypertension, substance use disorders, and suicidal tendencies [22–28]. In addition to these factors along with the fact that insomnia results in decrement in life quality and a greater risk for falls and accidents, it is suggested the therapy should be specific for treating the insomnia once diagnosed, even if it occurs along with psychiatric or physical conditions [29, 30]. More than 80% of adults are affected every year by occurrence of short-term or acute insomnia. Chronic insomnia (more than 3 months of time span) is characterized by trouble in onset of sleep, inadequate sleep, or nonrestorative sleep resulting in complaints of daytime sleepiness, irritability, exhaustion, or problem in focusing and carrying out day-to-day work [31]. For individuals meeting the symptomatic basis for insomnia, various scientific therapies can be approached including nonpharmacological as well as pharmacological therapies [32–34]. The suggested first-line treatment for insomnia is nonpharmacologic, like sleep restriction, relaxation training, and stimulus control. Drugs used for the treatment of insomnia are categorized as sedatives, hypnotics, drugs inducing sedation as a side effect, the drugs used in the treatment of insomnia-inducing sleep diagnoses like restless leg syndrome (RLS), and medicines regulating the sleep-associated circadian neuroendocrine system [35–37]. The impact of this disorder on public health with regard to frequency, morbidness, and effects on health and quality of life strictly requires its effective diagnosis and treatment in the clinical practice. This objective of this article is to review the diagnosis and treatment strategies for insomnia [34].

## 2. Pathophysiology of Insomnia

Various factors usually responsible for insomnia are psychological, physical, biological, environmental, and their interactions. Insomnia is frequently believed to be a disease related to hyperarousal [38], or enhanced cognitive, somatic, and cortical activation [39]. Patients with insomnia may encounter physiologic hyperarousal in both peripheral and central nervous systems. Hyperarousal can also be indicated as emotional and cognitive processes, with few theories recommending that affective and cognitive hyperarousal at sleep time may result in both acute and chronic insomnia [40]. Hyperarousal can be determined by measuring EEG, variability in heart rate, raised cortisol, or even self-report.

### 2.1. Genetics Involved in Sleep and Insomnia

Sleep and wake attributes, for example, sleeping time and duration, are hereditary [41] and controlled by various genes [42]. Involvement of genetic mechanisms in the etiology of insomnia is also implicated in animal and human investigations. Volunteer's gene assessment has recognized gene variants that might be engaged in the pathophysiology of insomnia, such as PER3^4/4^ [43], homozygous Clock gene 3111C/C Clock [44], Apo*ε*^4^ [45], short (s-) allele of the 5-HTTLPR [46], and HLA DQB1∗0602 [47]. A study based on genomes revealed various single-nucleotide polymorphisms considerably related to insomnia symptoms [48]. The most considerable single-nucleotide polymorphisms found within genes are associated with stress reactivity (e.g., STK39, USP25, and MARP10), neuroplasticity (e.g., ROR1, PLCB1, EPHA4, and CACNA1A), mental health (e.g., NPAS3), and neuronal excitability (e.g., GABRB1 and DLG2) [48].

As a whole, the abovementioned evidences suggest considerable heritability and involvement of multiple gene in the pathophysiology of insomnia. Genes most consistently found to be related to insomnia are genes linked to sleep-wake processes, arousal regulation, and brain functioning. The unpredictable interplay of these genes is responsible, at least to a limited extent, for the heterogeneity discovered in insomnia indications and outcomes. Future hereditary investigations with thorough evaluation of health and sleep history of individuals with chronic insomnia problem may additionally refine our comprehension of hereditary components associated in the characteristics and development of insomnia.

### 2.2. Molecular Mechanisms of Sleep and Insomnia

Various substances regulating sleep are connected to sleep regulation and circadian rhythmicity. In spite of perceiving the oversimplification [49], it is contended that endogenous substances are classified mainly as wake-suppressing/sleep-promoting (such as *γ*-aminobutyric acid [GABA], prostaglandin D2, serotonin, adenosine, and melatonin) and sleep-suppressing/wake-promoting substances (like orexin, catecholamines, and histamine). A smaller number of molecular investigations were performed on insomnia, and only a few molecules have been focused (e.g., cortisol and GABA) [50]. Results are mixed across investigations, without any steady pattern for a particular kind of molecule (wake versus sleep-inducing) has arisen. In spite of conflicting evidence, findings have mainly been deciphered with reference to hyperarousal model. For instance, raised [51] and decreased [52] levels of GABA in the occipital cortex of individuals with insomnia were found to be compatible with the hyperarousal hypothesis of insomnia. Nevertheless, sleep regulatory molecules link with one another unpredictably, and large number of their impacts are reliant upon the milieu of the brain state, i.e., they are state-dependent. The abovementioned parameters make it unpredictable that all instances of insomnia can be described by changes in any one kind of molecule (e.g., hyperarousal-related). Further refined conceptualization states that chronic insomnia develops from fragmentation of the alternating rhythms of sleep-regulatory and wake-promoting molecules in the brain [53].

Humans respond with different patterns in body temperature alterations, feeding, sleeping, and other biological functions to cycles of light and dark. The light signals received by the retina are translated by the pineal gland into a language understandable to the rest of the body, for example, via the synthesis of the hormone melatonin generally produced and released at night and thus regulating the metabolic activity of the body during sleep. The synthesis of melatonin and its release in the pineal gland are also regulated by another hormone norepinephrine. Norepinephrine functions by binding to its receptors in cell membranes. Earlier, it was thought that all these norepinephrine receptors acted independently of other proteins. However, in the recent investigations, it was found that this is not the case. Actually, these receptors collude with other dopamine receptors to form “heteromers.” Now, if dopamine binds with its receptors, it results in inhibition of norepinephrine effects, i.e., a reduction in the production of melatonin and its release. Incidentally, it was found that these dopamine receptors become visible only in the pineal gland near to the end of the night, as the dark phase diminishes. Thus, it was concluded by the researchers that the heteromer formation is an effective mechanism to halt melatonin production as the day starts and to “wake up” the brain. “These findings are fascinating as they revealed a mechanism by which dopamine, generally raised at times of stimulation, can directly hinder production and release of a hormone, melatonin, responsible for inducing drowsiness and prepares the body for sleep” [54].

## 3. Classification of Sleep Disorders

The primary classification of sleep disorders, the Diagnostic Classification of Sleep and Arousal Disorders, published in 1979 [55], classified sleep disorders into symptomatic classes forming the ground for the current classification systems. The International Classification of Sleep Disorders (ICSD) was first published in 1990. The ICSD classification, prepared mainly for epidemiologic, diagnostic, and at the point, research intention, has been broadly utilized by researchers and has permitted greater international correspondence in research related to sleep disorders [56]. The ICSD underwent minor updates and alterations leading to version 2 (ICSD-2) in 2005. The ICSD-2 classified the 81 major sleep disorders in eight significant classes. On the same basic outline as the ICSD-2, ICSD-3 was prepared, identifying seven major categories as follows [57]:

1. Insomnia
2. Sleep-associated breathing disorders
3. Central disorders of hypersomnolence
4. Circadian rhythm sleep/wake disorders
5. Parasomnia
6. Sleep-associated movement disorders
7. Other sleep disorders

The disease insomnia can be classified as primary and secondary insomnia. Though the etiology of primary insomnia includes both intrinsic and extrinsic factors associated, yet they are not viewed as being secondary to another problem. Secondary insomnia mainly occurs due to manifestations of a psychiatric or medical disease, another sleep problem, or drug addiction [58]. Though primary insomnia can appear in any individual, chronic insomnia mainly develops in a subgroup of people having fundamental inclination to insomnia [59]. Chronic insomnia is distinguished by indications that appear at least 3 times in a week for at least 3 months. Insomnias that remain for less than 3 months are termed as short-term insomnia. In few instances, individuals may display insomnia indications not fulfilling the basis for short-term insomnia and so require different kinds of therapy and is termed as other insomnia [60].

Though insomnia can be distinguished by various methods, most diagnoses can be classified as one of two categories [61]:

1. Sleep-onset insomnia is associated with problem in getting asleep. It mainly appears in individuals who have a hard time relaxing in bed, and also individuals whose circadian rhythm disturbs because of factors like jet lag or unpredictable work schedules
2. Sleep maintenance insomnia is associated with problem in continuing asleep after initial sleep onset. It mainly occurs in elderly people, along with persons who intake caffeine, tobacco, or alcohol before bed. Some diseases like sleep apnoea and RLS can also result in sleep maintenance insomnia

Few patients may have blended insomnia including both sleep-onset and sleep maintenance problem, and persons with chronic insomnia may feel that these indications shift over time.

## 4. Diagnosis

Insomnia is a sleep disorder in which a person is not able to complete his sleep due to various reasons which differ from person to person [62, 63]. The most common symptom generally witnessed is lack of concentration, loss of memory, decreased occupational, and social performance, aggression, irritation nature, and fatigue, etc. [4, 5, 35]. It is diagnosed on the basis of the trouble in falling sleeping, disturbance in continuing sleep, or awakening in early morning [5].

### 4.1. Diagnostic Tests for Insomnia

#### 4.1.1. Sleep Diary

A sleep diary is a patient's sleeping and awaking time record with concerned information, usually over a period of several weeks. It is made by person and physician to record the sleeping arising time [64, 65]. This is an important tool for diagnosis of insomnia by using sleep diary which helps in suitable treatment to the patients.

#### 4.1.2. Epworth Sleepiness Scale

The daytime sleepiness is measured by using Epworth sleepiness scale (ESS) with the help of short questionnaires. The possibility of falling asleep from 0 to 3 for eight different conditions is estimated by help of ESS. The scores for all the eight questions are calculated to obtain a single value. A score which lies in the range of 0–9 range is considered to be normal; while a score lying between 10–24 indicates sleep disorders [66].

#### 4.1.3. Actigraphy

It is used for recording human nonfunctional and functional activity cycles [67]. Actigraphy has a small altigraph part called a sensor of actimetry. It is used to measure gross motor action for a week or more. It is a wristwatch-like unit worn on the wrist [68].

#### 4.1.4. Athens Insomnia Scale (ASI)

It is most commonly used for diagnosing insomnia. It has 8 parameters in which 1 to 5 are related to nocturnal sleep, and 6, 7, and 8 parameters are associated with daytime dysfunction. ASI parameters include sleep promotion, awakening during the night, final awakening, total sleep duration, sleep quality, wellbeing during the day, functioning capacity during the day, and sleepiness during the day [69, 70] (as shown in Table 1).

#### 4.1.5. Laboratory-Based Polysomnography (PSG)

It is used for the diagnosis of obstructive sleep apnoea syndrome (OSAS). It can be used for diagnosing various sleep disorders like periodic limb movement disorder, chronic insomnia, narcolepsy, and REM sleep behaviour disorder [71].

#### 4.1.6. ICSD-3 Technique

It is most commonly used classification system for sleep disorders, with diagnostic criteria for insomnia disorder summarised [61, 65, 66]. Individuals with chronic insomnia frequently suffer from concomitant mental disorders which do not get easily diagnosed [72].

#### 4.1.7. Multiple Sleep Latency Test (MSLT)

It is an insomnia diagnostic tool which is determined as the time elapsed from the start of a daytime nap period to the first signs of sleep called sleep latency. It is used to discriminate between physical tiredness and really extreme daytime sleepiness. Its focus is to find the mode of falling asleep in relation to REM sleep and other brain patterns. It is also used to recognize and distinguish among numerous sleep complications [73].

### 4.2. Clinical Assessment of Insomnia

#### 4.2.1. Chief Complaint

Insomnia is caused by various variables factor. These complaints can be completely separated into nighttime and daytime. The chief complaint of patient should be assessed about initiation as well as maintaining sleep, early daytime arousal, or just unrefreshing sleep is the chief complaint associated with insomnia [74].

#### 4.2.2. Examination of Medical Patient History

Patient medical history related to insomnia with short sleep period has been related to various diseases like type II diabetes, hypertension, and more terrible neurocognitive dysfunction. Consumption of alcohol is a usual maladaptive self-treatment approach by individuals having insomnia and can even contribute during sleep-maintenance problems. Therefore, alcohol consumption is being measured or evaluated and considered during treatment planning. Many times some drug interaction can also lead to insomnia so it must be evaluated in patient medical history in order to know whether the patient is suffering from any other disease (Table 2) [57].

#### 4.2.3. Sleeping History

The sleeping history incorporates sleep and awakening schedule, bedtime schedule, night-time behaviour, and dysfunctioning during day. It is very important to determine the previous and current sleep history of patient. It is very useful for the treatment of insomnia patient. There are several questions which may help to determine sleeping history as follows [75].

*(1) Sleep and Wake Schedule*. It must be determined in work, school days, and weekends or vacations. A detailed note on schedule to bed, time to sleep, recurrence of night arousals, time to get back to sleep, time awakening in the morning, and time to get out of bed [76].

*(2) Bedtime Routine*. To confirm the clinical assessment of insomnia, it should be ensured that deficient sleep is not because of poor sleep environment. A detailed study on the bedtime routine is main part used during the treatment phase of insomnia [70].

*(3) Nocturnal Behaviour*. The many questions arise about nocturnal behaviour such as what the patient is doing while not sleeping at night? Are there other behaviours during night, like leg kicking or snoring which necessitates alternative or collateral diagnosis? Also, contribution from a bed partner can be useful. An individual who reported to be awakened the whole night, his bed partner will regularly notice longer duration of sleep, recommending there might be some sleep state misconception [77].

*(4) Daytime Functioning*. The daytime functioning can induce insomnia because person becomes tired, heaviness head, headache, and feel uncomfortable in daily routing. Hence, daytime function must be considered in clinical assessment of insomnia [78].

## 5. Management of Insomnia

Once a person is determined to be suffering from insomnia, and treatment might be started with one of various accessible mediations. They can be mainly classified into nonmedicated therapies and pharmacological treatment along with fewer herbal treatments [34].

### 5.1. Nonmedicated Therapies

Various nonmedication treatment regimens are available which have been tried and executed for the treatment of insomnia. In this section, the components assisting the nonpharmacological therapy having the greatest experiential background and most extensive use, i.e., cognitive behavioural therapy for insomnia (CBT-I) are reviewed [34].

#### 5.1.1. Sleep Hygiene and Cognitive Behavioural Therapies

Any patient suffering from insomnia should be asked for his sleep behaviours because sleep hygiene along with CBT-I can be used to treat insomnia without medications. Utilized in a number of patterns, CBT-I has been discovered to be successful in bringing down insomnia and improvement in sleep in a broad range of clinical populations [79–85]. Thus, the American College of Physicians has suggested this therapy to be the primary treatment for patients with insomnia [83] as this can avoid probable side effects and toxicities due to medicines and found to have long-lasting resolution in treatment of chronic insomnia that might give superior results as acquired from drug therapies [86]. Sleep hygiene signifies absence of required sleep accelerating approaches, drugs, dietary components, and environmental factors that can cause insomnia. Various drugs inducing insomnia are activating agents of prescription and abuse, weight loss preparations, nicotine, and caffeine.

Sleep hygiene can improve sleep by affecting sleep latency and time awake after sleep onset, total sleep time, and slow wave sleep; and influencing REM sleep. Some of the sleep hygiene recommendations are exercise, limiting caffeine, avoiding alcohol, food and liquid intake, bedroom environment, etc. The various advantages of exercise can decrease in sleep latencies and time awake after sleep onset; increase in total sleep time and slow wave sleep; and a slight delay, and minimal reductions, in REM sleep [87].

Caffeine intake before bedtime results in prolonged sleep latency, reduced total sleep time, and decrease in slow wave sleep; usually in a dose–response manner [88]. Alcohol being a sedative can be an appealing hypnotic [89]. Ebrahim and colleagues [90] reviewed that though alcohol intake can reduce sleep onset latency and a more consolidated first half sleep, it is also related to an increase in disturbed and fragmented sleep in the second half of sleep. Furthermore, though low and moderate doses show little effect on REM sleep, high doses can remarkably reduce REM, mainly in the first half of the night.

Sleeping hungry or eating a heavy meal before sleep is generally not recommended, and also, liquid intake should be minimized in the evening (2–3 h before bedtime). Obviously, it suggests that trying to sleep can become more challenging if a person is digesting a heavy meal, particularly if it contains high levels of fat or spice. Moreover, sleeping hungry may sometimes result in decrease in blood sugar in the night, keeping the individual awake. Excess amount of liquid during the evening may result in increased urination resulting in increased nocturnal awakenings [91].

The sleep environment should be quiet, dark, cool, and comfortable, and more recently, free from electronics. In a study on Europeans, it was recommended that a hot bedroom or an uncomfortable bed can result in nonrestorative sleep [92]. Environmental modifications such as eye masks, blackout blinds, earplugs, use of suitable sleepwear, and bedding and new mattresses are encouraged under this recommendation. Both excessively hot and colder environments in the bedroom can negatively influence sleep. An ideal sleep temperature between 60°F/15.6°C and 67°F/19.4°C is suggested by the National Sleep Foundation [93]. Lights in the bedroom may prevent a person from sleeping or make him awake earlier than desired because of its influence on the circadian system and the melatonin suppression. Bedroom noise has been the most studied bedroom environmental factors [94]. The findings are clear in that excessive noise disrupts sleep and subsequent daytime performance, even if the person does not recall awakening during the night [94]. The WHO suggests night-time noise should be below 40 dB [95, 96].

CBT incorporates sleep hygiene in the utilization of sleep facilitating cognitive and behavioural approaches for the treatment of insomnia [97]. When CBT is provided by trained personnel for several extended appointments, it proved its usefulness in the treatment of chronic insomnia [98]. Though insomnia therapy can be restricted to the use of CBT and hygiene, yet this approach has some clear constraints. But these behavioural approaches are not recommended for treatment of acute and short-term insomnia with restricted value in treatment of insomnia with comorbidities. CBT-I needs patient interest and their efforts, but as a clinical approach, it may not be available to all affected persons because of both expense and constraints in provider availability [99]. Even if judiciously used, it may not provide relief to every patient [100]. CBT is generally given for about 4-7 sessions. However, still it is not clear that how many sessions are required for optimal benefit, it was found that less than four sessions will not provide the desired response [80, 101].

*(1) Educational Components of CBT-I*. Though most of the patients of insomnia are well known regarding the various behaviours that are included under the category of sleep hygiene, it is still necessary to educate them regarding the suitable conditions. These are the significance of providing a favourable sleep environment by maintaining darkness, peace, and coolness in the bedroom. Patients need to remember to avoid consuming sleep-disturbing substances, like alcohol, nicotine, and caffeine, especially before bedtime. Similarly, strenuous exercise should not be done at least three to four hours before bedtime. In addition, a relaxing schedule can be useful in preparing an individual for sleep including preventing arousing activities like exposing to bright light (like laptop screen), which can have negative impact on someone's circadian rhythm [34].

*(2) Behavioural Components of CBT-I*.

*Stimulus Control*. Awakening due to some conditions is one of the major parameter involved in the pathogenesis of insomnia. Vicious circle of the bedroom/bed and episodes of physiologic awakening, fright, nervousness, and irritation prompts the bed serving as the known indication or conditioned stimulus for awakening, which is not compatible with sleep onset and maintenance. To get out of this conditioned stimulus, individuals are advised to keep themselves away from the bedroom and bed if not feeling sleepy and sit quietly somewhere until the sensation of sleepiness returns. Also, at sleep time, the patients are advised to avoid going to bed till they feel sleepy. The bedroom and bed should be strictly used for sleeping and sex, i.e., the patients should not do any other activities in bedroom, like reading or watching television. At last, patients are advised to get up at the same time every morning, seven days/week, and leave the bed within 10 to 15 min after arousing [34].

*Sleep Restriction*. Tendency of the patients to remain in bed for some more time duration is the other common reason responsible for the evolution and preservation of insomnia. In support of this, a reasonable answer has been given that the patients want to “get” sleep at whatever point they can. But again, this excess duration in bed brings about conditioned arousal and interrupted sleep. To adequately do this procedure, patients should be given sleep diaries for at least one week (however, 2 weeks are favoured), thereby reducing the patient's time in bed to the total sleep hours. For example, if a patient's diary reported six hours of average total sleep time however a period of nine hours in bed (bedtime 9 pm and awakening time 6 am), then a new sleep timetable should give a time of six hours in bed (bedtime 12 pm and awakening time 6 am) [102].

Particularly, patients are advised not to go for sleep until the newly recommended sleep time and only when sleepy. The patient's chronotype should be taken into account while finalising his sleep opportunity window. A minimum time of 5 hours in bed has been mentioned in the literature because of safety reasons related to sleep restriction (for example, somnolent driving and cognitive deficits) [102]. Also, sleep restriction may intensify comorbidities. For example, sleep restriction has been found to precipitate mania in individuals with bipolar disorder, lower seizure thresholds, and increase pain sensitivity [100, 103, 104].

Sleep diaries should be filled by the patients throughout the therapy. Their schedules regarding timings in bed must be evaluated in all successive CBT sessions, and each session should be scheduled after every one to two weeks. The physicians can determine patients' average sleep efficiency from the sleep diaries, calculated as the percentage of time a patient is asleep given his/her time in bed. An average sleep efficiency of 85% or more is preferred as a measurement for “good” sleep quality and an edge to be reached before modifying the time in bed proposal. Whenever it is confirmed that the sleep efficiency of patient is acceptably significant, the physician can increase the time in bed, especially by modifying the recommended bedtime by 15 min after each session and following the patient's improvement in subjective sleep quality and daytime sleepiness.

Sleep restriction is the part of CBT-I that mainly suffers due to patient's refusal. If a patient is not willing or unable to complete the recommended time in bed, sleep compression may be utilized. This approach comprises of gradual decrease in time in bed over time so that the original recommended time in bed can be met and might be more compatible with patients, especially those with higher nervousness about losing further sleep opportunity.

*Relaxation and Paradoxical Intention*. These behavioural approaches supplement sleep restriction and stimulus control by furnishing the patient with techniques to decrease arousal before bedtime and in case of awakenings during nighttime. Relaxation techniques vary, however, it includes the tensing and relaxing of muscle groups, diaphragmatic breathing, and potentially visual symbolism. Paradoxical approach is based on the concept that sleep onset is suppressed due to nervousness about getting asleep. Utilizing this approach, patients are advised to remain awake as long as possible, resulting in faster onset of sleep due to reduced nervousness.

*(3) Cognitive Components of CBT-I*. Abnormal thoughts and assumptions regarding sleep are especially considered throughout the therapy. Sleep-related concerns should be significantly attended by the clinician, as they will induce unsuitable behaviours that extend insomnia. These sleep-related worries include unrealistic assumptions regarding sleep and cataclysmic thoughts about the effects of sleep loss. One way for combating cataclysmic thinking is by assessing evidences from the experiences of patient. For example, if a patient thinks that a low sleep time during night will make him/her incapable to perform effectively in his/her work, then, a physician should ask the patient recognizing cases when he/she was able to perform adequately in spite of a poor sleep during the night. Moreover, equipping patients with techniques to decrease stress at bedtime can be useful.

Second approach, known as a constructive worry exercise, in which patients are required to make a list of at least three problems in the early evening which they think will make them awakened at night. The patients should mention a solution for every problem. The diary should be closed and kept away, and, now if patients wake up at night, they are advised to remind themselves that they have already resolved that issue at their “problem-solving best” (i.e., not in the midnight) [34].

### 5.2. Pharmacological Therapies

Irrespective of the kind of treatment given, the therapy of chronic insomnia has two key goals: improving quantity and quality of sleep and decreasing daytime impairments. Initial treatment strategies generally involve at least one behavioural mediation, like relaxation therapy or stimulus control therapy. In case pharmacotherapy becomes necessary, a particular medication within a class should be chosen as per the following directions: (1) pattern of symptoms, (2) objectives of therapy, (3) previous therapy responses, (4) cost, (5) patient compliance, (6) contraindications, (7) the accessibility of other medicines, (8) comorbidities, (9) possible adverse effects, and (10) simultaneous medication interactions [10].

This section reviewed the characteristics of all of these pharmacological agents (orexin antagonists, “z-drugs,” benzodiazepines, selective histamine H1 antagonists, nonselective antihistamines, melatonin receptor agonists, antipsychotics, antidepressants, and anticonvulsants) (Table 3) and compiled the available evidences related to their efficiency and safety on the basis of which clinical decision can be made.

#### 5.2.1. Benzodiazepines

These drugs act by modulating the *γ*-aminobutyric acid (GABA) type A receptors in neurons causing activation of chloride channels that produces Cl^−^ ion influx and cell hyperpolarization [105]. GABA works as an inhibitory neurotransmitter in the CNS, diminishing neuronal excitability. By stimulating the GABA receptors, benzodiazepines cause sedation, diminished nervousness, retrograde amnesia, and muscle relaxation [18, 106]. Though various benzodiazepines are utilized in insomnia treatment, only five (triazolam, flurazepam, temazepam, quazepam, and estazolam) are authorized by the U.S. FDA for insomnia. A few significant differences should be considered while a benzodiazepine is being prescribed for insomnia, like onset, duration of action, and metabolism. Rate of metabolism is especially significant in individuals with compromised kidney and/or liver function or elderly patients as these drugs may cause bioaccumulation, leading to adverse effects like daytime sleepiness, loss of coordination, and memory impairment. In addition to these effects, long-term use of benzodiazepine will disrupt the sleep quality by deforming the sleep architecture and declining deep sleep duration, thereby justifying the point that patients taking benzodiazepines for long term reported much more tiredness than self-reported good sleepers [2].

There is a high risk of emerging physical dependence to benzodiazepines [107]; extreme withdrawal indications after cessation has been reported by 15% to 40% of chronic users [108]. Even there are cases, when patients often reported increased nervousness and rebound insomnia after only a few weeks of benzodiazepine treatment [109]. These drugs have shown contraindications during breastfeeding and pregnancy. Benzodiazepines should be avoided in persons having chronic pulmonary disease and/or sleep apnoea as they may cause suppression of respiratory drive. Benzodiazepines are categorized as schedule IV drugs, due to their abuse potential.

#### 5.2.2. “Z-Drugs”

Zolpidem (Ambien), zaleplon (Sonata), and eszopiclone (Lunesta) are the most generally recommended category of medicines for insomnia, known as the z-drugs [110]. The efficacy of these drugs has been demonstrated in various trials including meta-analysis revealing that the sleep latency has been decreased by a mean value of 42 min versus 20 min for placebo [111]. The z-drugs bind in a similar manner as of benzodiazepines to the GABA-A receptor, resulting in cell hyperpolarization. But, the z-drugs as opposed to benzodiazepines bind with more specificity to particular subunits of the GABA-A receptor, basically focusing on the sedative effect of the receptor rather than the anxiolytic effect [112]. Various adverse effects of z-drugs especially in higher doses include gastrointestinal upset, dizziness, hallucinations, memory loss, and noninhibition. Sometimes, complex sleep-associated behaviours (for example, sleep eating, sleep driving) were found in persons using z-drugs in high doses; the patients should be informed regarding this risk initially while prescribing z-drugs [113–115]. The physician should also be known to the addictive potentials of these drugs as they may result in euphoria, anxiolysis, and stimulation in certain individuals, especially at higher doses [116]. These drugs have been utilized in the treatment of a wide range of sleep disorders without affecting sleep efficiency [107].

Zolpidem, an imidazopyridine used in the treatment of insomnia associated with problem of sleep latency and sleep maintenance, has also been used in the treatment of high-altitude insomnia and misalignment of circadian rhythm [117, 118]. Eszopiclone, a cyclopyrrolone drug due to its long half-life, has been utilized for the improvement of sleep latency, and it especially suits well for sleep maintenance [119]. But the only unfavourable aspect of eszopiclone is its unpleasant taste that influences almost 33% of patients at the maximum advised dose. A pyrazolopyrimidine drug, zaleplon, having very short half-life is recommended for the improvement of sleep latency but not sleep maintenance. All these drugs show a moderately distinct pharmacological profile, symptoms, efficacy, and adverse effects. These drugs can be prescribed during pregnancy just if the advantages are more than the risks associated. Zolpidem, eszopiclone, and zaleplon are categorized as schedule IV drugs due to their abuse potential.

#### 5.2.3. Melatonin Receptor Agonists

Melatonin is a hormone and is generally, secreted during the dark period of the day by the pineal gland. It plays a major role in maintaining the sleep-wake cycle, and disturbance in the melatonin release timings or diminished melatonin formation may result in insomnia and thus it is prescribed to many patients having insomnia. The issue becomes especially noticeable during shift work or when changing time zones. Production of melatonin also dwindles with age and might be partly accountable for the sleep problems encountered by elderly people [120]. Ramelteon (Rozerem) is a melatonin agonist authorized by the FDA for the treatment of sleep latency. Its efficacy is satisfactory with little adverse effects and the advantage of not habit forming [107]. A small investigation revealed that the drug has diminished the risk of hospital-related hysteria in elderly patients, bringing the rate from 32% to 3% in comparison to placebo [121]. This drug can be prescribed during pregnancy only if the advantages are more than the risks associated. For insomnia therapy, a sustained-release melatonin is considerably less costly and may be more efficacious as compared to ramelteon. A meta-analysis in 2013 revealed that melatonin in the dose range of 0.1 mg to 5 mg declined sleep latency by 7.1 min, enhanced total sleep duration by 8.3 min, enhanced overall sleep quality, and had a favourable adverse effect profile [122]. Since it is not governed by the FDA and purity fluctuates broadly among products, it is necessary to review product evaluations by third-party to assess the quality of the product.

The most widely recognized unfavourable effects of melatonin are headache, increase in reaction time, and may cause sedation during the day. It can be given to abuse-prone individuals with insomnia due to their nonabuse potential. Moreover, when given in high doses, it cans theoretical affect fertility as it is a hormone that controls reproductive function. Thus, ramelteon is not advised to those who are trying to conceive [123, 124]. It has a moderately benign adverse effect profile, among which the most generally revealed are nausea, fatigue, headache, and sedation. Because of its greater safety profile, it can be recommended for patients having problem in sleep onset only.

#### 5.2.4. Selective H1 Antagonists

Doxepin in the dose range of 3-6 mg is the exclusively very selective histamine H1 receptor antagonist that has been consistently investigated [30]. Though the major potent pharmacological effect of doxepin is H1 antagonism, it originally emerged as an antidepressant in the dose range of 75-150 mg/day [125]. Thus, with a decrease in the dosage, doxepin becomes a highly selective H1 antagonist [125]. Sedation during daytime is the most usual adverse effect disclosed in younger adults. On the other hand, elderly patients did not report any adverse effects with 3 mg of doxepin. And so, elderly patients with early morning arousal can be an especially appropriate group for treating with this drug. Due to its potent H1 antagonism, it can be recommended for patients having insomnia with allergy indications. Due to its nonabuse potential, doxepin can be given to individuals having difficulty in sleep maintenance and who are prone to addiction.

#### 5.2.5. Orexin Receptor Antagonists

“Orexins” was the title given to two peptides that were comparatively recently found to emerge from the neurons of the lateral hypothalamus and to provoke arousal/wakefulness [126–128]. Orexin receptor antagonists are the agents that promote sleep because of its potential to obstruct the wakefulness provoked by the orexins. The first orexin receptor antagonist that got the approval for insomnia treatment is suvorexant (Belsomra). It causes blockages of both kinds of orexin receptors (orexin A and B). Sedation during daytime is the most usual adverse effect of suvorexant. Prior investigations revealed that some abuse potential is associated with this drug that is somewhat comparable to that of zolpidem. So, in patients predisposed to abuse, it is better to be avoided. It is an exclusive drug with therapeutic effects in the last third of the night without considerably enhancing morning sedation leading to strong therapeutic effect on sleep onset. Thus, suvorexant can be prescribed to those individuals having both difficulty in onset of sleep and arousal in early morning. However, it is not advised as a first-line therapy for insomnia due to its higher cost and abuse potential.  Table 4 compiles significant orexin receptor antagonists that were developed/abandoned or are in developmental pipeline.

#### 5.2.6. Antidepressants

There are some drugs initially used for the major depression therapy are now also utilized for the insomnia treatment. This category of drugs also produces improvement in sleep by obstructing the receptors for neurotransmitters that cause arousal, e.g., acetylcholine, serotonin, histamine, and norepinephrine [30]. The most commonly recommended antidepressants for treatment of insomnia are 15 mg of mirtazapine, 10-75 mg of doxepin, 50-150 mg of trazodone, and 10-100 mg of amitriptyline [30]. The adverse effects caused by antidepressants for treating insomnia may vary. Daytime sedation is commonly caused by all, while some may cause orthostatic hypotension. The major adverse effects of mirtazapine are enhanced appetite/weight gain and sedation. The amitriptyline and doxepin (25-50 mg) may cause cognitive impairment, blurred vision, dry mouth, arrhythmias, constipation, increased appetite/weight gain, and urinary retention [30]. Trazodone is responsible for causing adverse effects such as sedation and orthostatic hypotension and may induce priapism [30].

Due to their nonaddictive potential, all these drugs can be given to patients with an inclination to drug abuse. These drugs can also be prescribed to patients in whom the commonly used therapy failed or have associated conditions like nervousness, mood, and pain difficulties, due to their wide pharmacological outcomes [30]. Patients suffering from glaucoma, urinary obstruction, and cognitive impairment should be given doxepin and amitriptyline cautiously. All these drugs are troublesome in individuals with bipolar depression, due to the fear of accelerating mania [138].

#### 5.2.7. Nonselective Antihistamines

Doxylamine and diphenhydramine are the two major nonselective antihistamines that are usually recommended for insomnia therapy. However, these are prescribed only for the therapy of pregnancy-related sleeplessness. Both of these drugs show clinically relevant M1 muscarinic cholinergic antagonism in addition to H1 antagonism. The major adverse effects of these drugs are dizziness, sedation, blurred vision, cognitive impairment, psychomotor impairment, dry mouth, weight gain, urinary retention, and constipation. More uncommon side effects of doxylamine reported are coma and rhabdomyolysis, whereas that of diphenhydramine is agitation and insomnia [139].

Due to their insignificant addictive potential, these drugs can be recommended for using in drug abuse-prone insomnia patients. These are also suitable for insomnia patients having upper respiratory infections or allergic symptoms. These medications should not be given to patients with urinary retention, chronic obstructive pulmonary disease, asthma, reduced gastrointestinal motility, and closed-angle glaucoma.

#### 5.2.8. Antipsychotics

These are the category of drugs mainly used for treating the psychotic states that are occasionally also utilized for the treatment of insomnia, but at a dose lower than that usually used in the treatment of psychotic patients [30]. These drugs produce sleep-improving effects owing to their broad antagonistic effect on arousal-provoking neurotransmitter receptors, like serotonin, histamine, dopamine, adrenergic, and cholinergic receptors. The antipsychotic drugs that are recommended in the treatment of insomnia in clinical practice are risperidone 0.25-6 mg, olanzapine 2.5-20 mg, and quetiapine 25-250 mg. The major adverse effects of these drugs include agitation, sedation, tachycardia, orthostatic hypotension, dizziness, increased appetite/weight gain, dry mouth, akathisia, and constipation. Really concerning, however, undeniably more uncommon, is the fear of tardive dyskinesia. In patients suffering from dementia, the high possibility of cerebrovascular episodes must also be considered.

Because of their nonaddictive potential, these drugs can be recommended to patients with an inclination to drug abuse. However, these drugs are most appropriate for insomnia arising in persons suffering from bipolar or psychosis diseases. Precautions must be taken, while using these medications in patients with closed-angle glaucoma, urinary retention, dementia, constipation, and hypotension or at risk for myocardial infarction.

#### 5.2.9. Anticonvulsants

There are some drugs initially developed for the seizure therapy that are now also used for the insomnia management. Examples are pregabalin and gabapentin, and they produce therapeutic effects in insomnia by decreasing the discharge of norepinephrine and glutamate by acting on the alpha-2-delta subunit of N-type voltage-gated calcium channels [140, 141]. A clinical trial study in patients suffering from occasional sleep disturbance revealed that gabapentin in a dose of 250 mg at bedtime enhanced total sleep duration by 64 min on 1^st^ day and 46 min on 28^th^ day of the trial [142]. Pregabalin and gabapentin also showed therapeutic effects on sleep disturbance in patients suffering from generalized anxiety disorder, pain, epilepsy, and restless legs syndrome [143–145].

The major adverse effects of pregabalin are dry mouth, dizziness, sedation, appetite increase, and cognitive impairment whereas for gabapentin includes ataxia, dizziness, sedation, and diplopia. Gabapentin does not have any abuse potential, whereas pregabalin showed some significant addictive potential [146]. These drugs can be recommended to insomnia patients also suffering from pain, RLS, or partial seizures. Pregabalin can also be used in patients with alcohol use disorder for the treatment of insomnia [147, 148]. In case of patients with impaired renal function, both these drugs should be avoided.

## 6. Treatment Considerations for Specific Populations

### 6.1. Circadian System Disturbances

Sleep disorders associated with circadian rhythm are due to an imbalance between about 24 h endogenous circadian rhythm and the “normal” 24 h day/night cycle. In patients with circadian disturbance, melatonin may work as a hypnotic and can be a beneficial supplement in the therapy [149]. Ramelteon, a synthetic analog of melatonin, is available for prescription. Light exposure and melatonin have been proved to be particularly effective for the treatment of Delayed Sleep Phase Syndrome (DSPS) mostly occurring in young adults and adolescents [149]. Patients with this condition have problem getting sleep at the desired bedtime and have delayed sleep between 2 am and 6 am and afterwards if their way of living allows, sleeping roughly for about 8 h, waking up in between 10 am and 2 pm. Patients suffering from DSPS frequently experiences long-term deficient sleep duration with all its daytime effects. Therapy includes bright light exposure at the right time in the circadian phase response graph.

Therapy includes providing 10,000 lx of light for half an hour on arousal and timely administration of melatonin in the early evening 3–6 h before sleep time (prior to dim light melatonin onset (DLMO)). Advanced sleep phase syndrome (ASPS) is the mirror image of DSPS in individuals having onset of sleep and arousal both few hours before time than required with the total sleep duration remaining almost usual. ASPS is more uncommon as compared to DSPS and mostly occurring in middle-aged to elderly patients. Similar therapy as that for DSPS has been applied with the timing of therapy intended to retard rather than forward the circadian rhythm. Persons working in shift are recommended melatonin for shifting the circadian rhythm of the workers as desired. If given prior to bedtime, it can improve the sleep quality in the early morning [150, 151].

### 6.2. Restless Legs Syndrome (RLS) and Periodic Leg Movement Disorder

RLS is a typical neurological condition distinguished by the desire to move, especially the legs, occurring mainly at rest in the evening or bedtime. The fundamental characteristics for diagnosing RLS are as follows:

1. Desire to move the legs, generally followed by awkward and uncomfortable sensations in the legs
2. Desire to move or uncomfortable sensations start or deteriorate with rest or inactiveness
3. The desire to move or uncomfortable sensations are somewhat or completely relieved by movement
4. The desire to move or uncomfortable sensations are unacceptable in the evening [152]

About 3/4 of the patients with RLS suffer from sleep disturbance and grievances of diminished life quality [153]. Most of the RLS patients suffer from recurrent periodic limb movements (PLMD) on polysomnogram. The PLMD/RLS has a hereditary basis and rises with age so that over 30% of individuals having age more than 80 years may fulfil the diagnosis criteria. PLMD/RLS is also commonly found in patients with low serum ferritin levels (<50), children with AD/HD, in patients on medications like antihistamines, antiemetics and antidepressants, and in patients having renal failure [154]. The therapy of the sleep disturbance, basically onset of sleep, depends primarily on therapy of RLS instead of treatment of resultant insomnia. The primary initial medications for RLS treatment belong to dopaminergic agonists. Both ropinirole and pramipexole have been approved by FDA for RLS but in low doses as compared to that required in Parkinson's disease. Ropinirole is given in the range of 0.25 to 4 mg and pramipexole at 0.125 to 2 mg. Pramipexole may initiate remarkable drowsiness along with sleep attacks in certain patients [155]. Earlier, benzodiazepines like clonazepam have been used, but also temazepam.

### 6.3. Insomnia Associated with Sleep Apnoea and Its Therapy

A significant percentage of population has been affected with obstructive sleep apnoea (OSA) that causes daytime sleepiness. Both the severity of apnoea and the degree of daytime sleepiness influencing waking capacity can be adversely influenced by using sedatives concomitantly—especially ethanol and opiates [156]. In a subgroup of OSA patients, breathing disturbance results in intermittent sleep and insomnia. The therapy of OSA with positive airway pressure (PAP) can enhance quality of sleep for these patients [157]. But, for some other individuals, PAP therapy may intensify sleeplessness (especially in patients with co-morbid PTSD) [158].

### 6.4. Comorbid Psychiatric Disorders

Major depression, psychotic disorders, anxiety disorders, bipolar mood disorder, and Alzheimer's disease are the various psychiatric disorders generally comorbid with insomnia. A range of 50–75% of incidences of insomnia with these diagnoses was estimated [159]. The diagnosis of depression in which depression and insomnia have a bidirectional or circular correlation is the most well-known psychiatric association [160]. Several investigations revealed the risk of developing depression in insomnia patients. A wide investigation on young volunteers for a time span of 20 years predicted that 14 days of insomnia or prolonged further will cause major depressive episodes and disorders [109]. Reoccurring insomnia are the indications that an individual in remission from his depression can be at risk of a relapse [161]. Long-term insomnia issues can result in the continuance of depression. The problem is of specific significance in light of the considerable rate of residual sleep disruption in patients who were otherwise effectively treated for depression [111]. The relation between depression and insomnia will get complicated by the certainty that most of the commonly used antidepressants, particularly the selective serotonin reuptake inhibitors (SSRI's), can result in sleep disruption [162]. Individuals diagnosed with a psychiatric disorder and insomnia, therapy includes those also prescribed for primary insomnia, either cognitive behaviour treatment (behavioural and psychological), pharmacologic treatment, or a blend of both.

Eszopiclone along with fluoxetine has been investigated in persons with major depression [163]. The combination was well tolerated and showed quick improvement in sleep along with a faster and greater antidepressant response. This does not propose an antidepressant effect of eszopiclone yet rather recommends that improvement in sleep has a favourable effect on depression. These findings provide support for the comorbid strategy for the therapy with two drug moieties as opposed to the conventional strategy of waiting for insomnia to recover because of treatment of depression. Combined therapy involving antidepressants and CBT for insomnia also suggested that the combined therapy is better than the antidepressants alone both in regards of depression outcome and insomnia outcome [164].

### 6.5. Comorbid Pain

Most of the patients have poor sleep due to chronic pain [165]. Pain can be a major symptom of most of clinical diseases however most frequently rheumatologic disorders, headache, and cancer. Chronic pain and sleep disturbance develop a cycle of pain resulting in inadequate sleep which further resulted in greater pain [166]. Comorbid pain can be managed by diagnosing the sleep problem, maintaining the sleep hygiene, and applying CBT approach followed by use of pharmacological agents for both insomnia and pain [98]. In case of rheumatologic diseases, treating sleeplessness with hypnotics or sedative antidepressants not only enhances sleep but also improves pain tolerance.

### 6.6. Other Comorbid Medical Conditions

Patients having respiratory disorders frequently have disturbed sleep. COPD patients often have interrupted sleep [167]. This can be improved by providing oxygen if hypoxia is the reason for the issue. Though OSA generally causes daytime sleepiness, it can also cause disturbance in sleep too [168]. Approximately one-third of asthma patients who are ineffectively treated have asthma attacks during night that may interrupt their sleep resulting in daytime sleepiness. Individuals with gastroesophageal reflux frequently get their sleep disturbed due to the reflux. Also, reflux can precipitate asthma attacks in weak patients. Individuals having last-stage renal disorders experience a number of sleep problems like sleep apnea, insomnia, and a high occurrence of secondary RLS with a high predominance [169]. Menopause can be related with sleeplessness which can be treated with hormones but also requires a hypnotic [170]. Parkinson's disease is related with crucial insomnia, as in case of gastrointestinal disorders causing reflux and/or pain, enuresis and nocturia, and various sleep-related issues such as narcolepsy [3].

## 7. Herbal Treatments

### 7.1. Valerian

Since ancient Greek and Roman times, Valerian, a herbal product comprised of the roots of *Valeriana officinalis*, has been utilized for the insomnia therapy [171]. It works by interacting with GABA-ergic neurotransmission, resulting in sedation [172]. Though few investigations have reported that valerian is valuable for the insomnia therapy, others have not [173–175]. Evaluation of the accessible clinical results are complicated by utilization of variable amounts and different sources of valerian, small sample sizes, different results obtained, and due to high rate of withdrawal [171]. As a whole, the evidences for valerian as an alternative for insomnia therapy remains uncertain, and it is not advised to use in insomnia patients [114].

### 7.2. Kava

Kava, a herbal product obtained from a shrub (*Piper methysticum*) and cultivated in the Pacific islands, works by acting on both BZD and GABA binding sites, producing antispasmodic, anticonvulsive, central muscular-relaxant, and sedative impacts [176]. Kava-containing medications are utilized as alternative treatment for restlessness, stress, and nervousness—significant reasons for long-term insomnia [176]. Notwithstanding, like various herbal products, kava is not suggested for the chronic insomnia therapy due to absence of clinical efficacy and safety data [114]. Kava-containing products were found to cause severe liver injury as warned by the FDA in 2002 [177].

## 8. Approaches to the Patient

For the insomnia treatment, behavioural interventions can be the best approach. In case, pharmacological mediation becomes essential, a customized approach depending on the insomnia type is recommended. In case, the first approach is not effective, an alternate drug from a similar category or a different category can be used. However, few patients can tolerate various sedative drugs, including a z-drug and sedating tricyclic antidepressant, utmost care must be taken while recommending such combinations. Due to the fear of respiratory depression, individuals having possibility of nocturnal hypoxia or sleep apnoea because of lung disorders must be examined by a sleep expert prior to prescribing sedatives. Benzodiazepines due to greater potential for abuse are usually not suggested. Even though benzodiazepines are to be used, they should be prescribed at the lowest possible dose for the shortest possible duration. Benzodiazepines, tricyclic antidepressants (except nortriptyline and low-dose doxepin), atypical antipsychotics, and z-drugs should not be given to patients with long-term nocturnal hypoxia, individuals with untreated sleep apnoea, and in older adults. Herbal drugs were also not proved to be very efficacious for the insomnia treatment [114].

## 9. Recent Updates and Patents on Insomnia Therapeutics

Lemborexant, a DORA that got approval in December 2019 for treating insomnia, was distinguished by trouble with onset of sleep and/or maintenance of sleep in insomniacs [178, 179]. The drug with a half-life of about 17-19 h diminishes sleeplessness and advances non-REM sleep without affecting REM sleep [180]. The drug was investigated in the SUNRISE trials [181] and reported a significant improvement in sleep onset and sleep maintenance in comparison to placebo. The commonly reported adverse effect in the SUNRISE trials was sleepiness [182, 183] without any respiratory issues. Volunteers with minor OSA did not encounter aggravating sleep apnoea, as determined by variations in peripheral oxygen saturation or apnea-hypopnea index [184]. Other orexin inhibitors in phase III clinical trials are seltorexant and nemorexant (daridorexant). Nemorexant is a DORA having 6 h of half-life. The commonly reported adverse effects were somnolence, diarrhoea, fatigue, and headache [185, 186].

Seltorexant is a selective orexin-2 receptor antagonist with 2-3 h of half-life. The exceptional mode of action produces hypnotic effects and also preserves normal sleep pattern along with decreased possibility of cataplexy [187]. In the phase II clinical studies, the drug was found to enhance sleep initiation and prolonged sleeping time. The drug is also under investigation for treating hyperarousal-associated insomnia in individuals with depression. Commonly reported adverse effects were dizziness, somnolence, and headache [188–190].

Various patents have been granted/published in this field including solid dosage forms, intranasal solutions, buccal aerosol sprays, and toothpaste, for the treatment of insomnia which are compiled in Table 5.

## 10. Conclusion

Insomnia is a typical and frequently crippling disease and is known to have remarkable adverse outcomes for physical health and well-being. For the treatment of this condition, fortunately, both behavioural and pharmacological therapies are available. Various treatments have been reviewed in this article to provide a resource for physicians, with the expectation that this would enhance the clinical management of insomnia. Currently, there is little customization of the most often utilized hypnotics depending on individual insomnia symptoms and comorbidities. Choice of therapy depending on the patient's particular symptoms and clinical either with dose titrations or by utilizing combination of drugs (within or outside the DORA class) may provide a type of customized insomnia treatment that until now has not been underlined. Significantly, since drugs in this category appears to have less addiction and minor rebound insomnia, the DORAS can be interestingly combined with CBT-I, which is generally viewed as an effective and enduring non pharmacological therapy for both primary and comorbid insomnia. With wide availability of novel modalities for CBT-I, like individual and group teletherapy, via web-based applications, and others, the role of customizable, titratable hypnotics might be exceptionally complementary. Nowadays when personalized clinical treatment is turning out to be progressively realistic, such advances may assist in keeping insomnia to bed.

## Figures and Tables

**Table 1:** Athens insomnia scale.

Assessment parameters	Sleep difficulty experienced at least three times per week			
	0	1	2	3
Sleep induction	No difficulty	Moderately delayed	Remarkably delayed	Extremely delayed or did not sleep at all
Arousals during night-time	No difficulty	Insignificant difficulty	Considerable difficulty	Serious difficulty or did not sleep at all
Final arousal before scheduled time	Not before	Little earlier	Remarkably earlier	Very insufficient
Overall quality of sleep	Satisfactory	Slightly satisfactory	Markedly satisfactory	Very unsatisfactory
Sense of well-being during daytime	Regular	Moderately declined	Remarkably declined	Extremely declined
Sleepiness during daytime	None	Moderate	Remarkable	Extreme

**Table 2:** Diagnostic management of insomnia.

Medical history and examination	Whether patient suffered with any neurological disorder such as amnesia, dementia, and epilepsy Previous and current somatic disorder Medication, alcohol, and caffeine Circadian makers
Psychiatric psychological history	Previous and current mental disorder Current and previous work condition Personality confliction
Sleep history	Circadian clock Sleep disorder history

**Table 3:** Comparison of commonly prescribed medications for the treatment of Insomnia.

Class of drugs	Dosage (mg)	Approximate *t*_1/2_ (h)	Approximate *T*_max_ (h)	Primary indication
Z-drugs				
Zolpidem, sublingual (Intermezzo)	1.75 or 3.5	2.5	1	Awakening in night
Zolpidem (Ambien)	5 to 10	2.6	1.6	Onset and maintenance of sleep
Zolpidem, extended release (Ambien CR)	6.25 to 12.5	2.8	1.5	Onset and maintenance of sleep
Eszopiclone (Lunesta)	1 to 3	6	1	Onset and maintenance of sleep
Zaleplon (Sonata)	5 to 10	1	1	Onset of sleep
Benzodiazepines				
Triazolam	0.125 to 0.25	1.5 to 5.5	2	Onset of sleep
Estazolam	0.5 to 2	10 to 24	2	Maintenance of sleep
Quazepam (Doral)	7.5 to 15	25 to 84	2	Maintenance of sleep
Temazepam (Restoril)	7.5 to 30	3.5 to 18.4	1.5	Onset and maintenance of sleep
Flurazepam	15 to 30	47 to 100	1	Maintenance of sleep
Tricyclic/quatracyclic antidepressants				
Doxepin (Silenor)	3 to 6	15	3.5	Maintenance of sleep
Mirtazapine (Remeron)	7.5 to 15	30	2	Not recommended
Amitriptyline	25 to 150	30	4	Limited use
Nortriptyline (Pamelor)	25 to 150	30	8	Limited use
Trazodone	50 to 100	10	1	Not recommended
Melatonin agonists				
Melatonin, controlled release	1	3.5	1.5	Onset of sleep
Ramelteon (Rozerem)	8	2.5	0.75	Onset of sleep
Antihistamines				
Hydroxyzine	50 to 100	20	2	Not recommended
Doxylamine	25 to 50	10	2.4	Not recommended
Diphenhydramine (Benadryl)	25 to 50	8.5	2.5	Not recommended
Orexin receptor antagonist				
Suvorexant (Belsomra)	5 to 20	15	2	Onset and maintenance of sleep
Anticonvulsants				
Pregabalin (Lyrica)	50 to 300	6	3	Limited use
Gabapentin (Neurontin)	300 to 600	6	2.5	Limited use
Antipsychotics				
Risperidone (Risperdal)	0.25 to 6	20	1	Limited use
Quetiapine (Seroquel)	50 to 400	6	1.5	Limited use
Olanzapine (Zyprexa)	2.5 to 20	30	6	Limited use

**Table 4:** Orexin receptor antagonists that were developed/abandoned/are in developmental pipeline.

Drug	Mechanism of action	Remark	Ref
Almorexant	An orexin antagonist that functions as a competitive receptor antagonist of the OX_1_ and OX_2_ orexin receptors	Development of the drug was abandoned in January 2011	[129]
Lemborexant	Dual orexin receptor antagonist (DORA) of the orexin OX_1_ and OX_2_ receptors	Approved by the FDA in December 2019 and released on June 1, 2020, by Merck.	[130]
Filorexant	DORA	Was under development by Merck for the insomnia treatment. As of May 2015, filorexant is no longer listed on Merck's online development pipeline.	[131]
Nemorexant	DORA	In April 2020, first phase III clinical trial for daridorexant has been approved for insomnia treatment.	[132]
SB-334,867	First nonpeptide antagonist emerged and is selective for the orexin receptor OX_1_, having about 50 times more selectivity over OX_2_ receptors	Studies in animals have shown sedative and anorectic effects.	[133]
SB-408,124	Is a nonpeptide antagonist selective for the orexin receptor OX_1_, having about 70 times more selectivity over OX_2_ receptors.	Enhanced oral bioavailability in comparison to the older OX_1_ antagonist SB-334,867.	[134]
SB-649,868	DORA	Developed by Glaxo SmithKline, The drug is currently in phase II development for insomnia.	[135]
TCS-OX2-29	Selective for the orexin receptor OX_2_, with an IC_50_ of 40 nM and selectivity about 250 times over OX_1_ receptors.	Nonpeptide antagonist developed	[136]
RTIOX-276	Binds selectively to the OX_1_ receptor (*K*_*E*_ = 8.5 nM) and lacks significant affinity for the OX_2_ receptor (*K*_*E*_ ≥ 10,000 nM).	Offering more specificity of action compared to nonselective orexin antagonists like almorexant.	[137]

**Table 5:** Patents granted/published for various dosage forms for treatment of Insomnia.

Patent no.	Country of filing	Composition/active ingredient	Major outcome	Publication/grant year	Ref.
8926991	USA	Botulinum toxin	For treating primary disorders of mood and affect with a neurotoxin, including depression, nervousness, and sleep disorders.	2015	[191]
US 8252809B2	USA	Zolpidem	Solid dosage form for treating midnight insomnia by administration of low doses (5 mg or less) of zolpidem or a salt thereof.	2012	[192]
US 8242131B2	USA	Zolpidem	Methods and compositions for treatment of midnight insomnia by administration of low doses (5 mg or less) of zolpidem or a salt thereof.	2012	[193]
US 8148393B2	USA	Zolpidem	A modified release zolpidem tablet is designed as a tablet-in-tablet dosage form.	2012	[194]
US 8034371 B2	USA	Zolpidem	Intranasal solutions of zolpidem for the prevention/treatment of insomnia or the therapy of neurological diseases.	2011	[195]
US 7914826	USA	Combination containing melatonin and other herbal supplements	Supplemental compositions and methods for inducing a restful night's sleep by rapidly inducing a person to fall asleep and to maintain sleep.	2011	[196]
US 7906154	USA	Combination containing melatonin and other herbal supplements	Supplemental compositions and methods for inducing a restful night's sleep by rapidly inducing a person to fall asleep and to maintain sleep.	2011	[197]
US 7655681B2	USA	Zonisamide	For treatment or prevention of obstructive sleep apnea (OSA) by the administration of a pharmacologically effective amount of zonisamide to a patient	2010	[198]
US 7632517B2	USA	Zolpidem	Zolpidem containing buccal aerosol sprays or capsules for faster absorption through the oral mucosa, leading to faster onset of effect for insomnia.	2009	[199]
US 6998112B2	USA	Sleep-inducing natural herb or hormone	A toothpaste for inducing sleep while simultaneously promoting intraoral cleanliness, which includes toothpaste base ingredients and at least one sleep-inducing natural herb or hormone and these are selected from the group consisting of chamomile, lemon balm, passion flower, and valerian, and the hormone melatonin.	2006	[200]
US 6703412 B1	USA	Melatonin	A method of treating sleeplessness in humans by administering an effective sleep-inducing amount of not greater than about 5 mg of melatonin	2004	[201]
